# Effect of Blade Outlet Angle on the Flow Field and Preventing Overload in a Centrifugal Pump

**DOI:** 10.3390/mi11090811

**Published:** 2020-08-27

**Authors:** Guangjie Peng, Qiang Chen, Ling Zhou, Bo Pan, Yong Zhu

**Affiliations:** 1 National Research Center of Pumps, Jiangsu University, Zhenjiang 212013, China; pgj@ujs.edu.cn (G.P.); 2221811017@stmail.ujs.edu.cn (Q.C.); zhuyong@ujs.edu.cn (Y.Z.); 2 Shandong Xinchuan Mining Equipment Co. Ltd., Jining 272300, China; xingyuan@sdxingyuan.com

**Keywords:** submersible pump, blade outlet angle, numerical calculation, external characteristics

## Abstract

The influence of the blade outlet angle on preventing overload in a submersible centrifugal pump and the pump performance characteristics were studied numerically for a low specific speed multi-stage submersible pump. The tested blade outlet angles were 16°, 20°, 24°, 28°, and 32°. The results show that the blade outlet angle significantly affects the external flow characteristics and the power curve can be controlled to prevent overload by properly reducing the blade outlet angle. Increasing the blade outlet angle significantly increases the low pressure area at the impeller inlet, which makes cavitation more likely. Therefore, β_2_ = 16° provides the best anti-cavitation flow field. Increasing the blade outlet angle also increases the flow separation near the blade working face, which increases the size of the axial vortex along the blade working surface, which rotates in the direction opposite to the impeller rotation and then extends towards the impeller inlet.

## 1. Introduction

Centrifugal pumps are widely used in many fields [1]. For medium and low specific speed centrifugal pumps, the shaft power increases sharply with increasing flow rate. Thus, high flow rates can easily overload the motor [2]. For micromachine fields, overload is one of the most common issues in small size pumps, such as the syringe pump and the heart pump, which has a strict requirement on the non-overload performance. Generally speaking, the maximum shaft power of low specific speed pump motors is generally greater than or equal to 1.2× the shaft power at the rated operating condition [3]. Stable operation of a pump without overload over the full head range [4] requires optimal design of the pump hydraulics. Therefore, it is of great theoretical and practical significance to study the effects of different parameters on the performance and reliably of low specific speed centrifugal pumps, especially to prevent power overload [5,6].

There have been many studies on the influence of the blade outlet angle on the flow characteristics of centrifugal pumps. Bacharoudis et al. [7] studied the effects of various blade outlet angles on the performance of centrifugal pumps to show that as the blade outlet angle increased, the pump performance curve became smoother and flatter. Nishi et al. [8] analyzed the radial thrust of two single vane pump impellers with different blade outlet angles and concluded that the larger blade outlet angle had better performance. Shigemitsu et al. [9] studied the effect of the blade outlet angle on the performance and internal flow conditions of a microturbine pump and showed that the maximum efficiency decreased with increasing blade outlet angle. Gölcü et al. [10] experimentally studied a deep well pump with splitter blades and related the number of splitter blades, the arrangement angle and the efficiency. Djebedjian [11] proposed a theoretical model for predicting the performance of split-blade centrifugal pumps. Pedersen et al. [12] and Byskov et al. [13] used large eddy simulations, laser Doppler velocimetry and particle image velocimetry to analyze the transient velocity fields in the flow path of a centrifugal pump impeller for design and off-design conditions. Barrios and Prado [14] experimentally and theoretically studied dynamic multiphase flow in a mixed flow submersible electric pump (ESP). Shojaeefard et al. [15] studied the effect of the blade outlet angle and impeller outlet width on the hydraulic performance of low specific speed centrifugal pumps. Studies have shown that increasing the impeller outlet width will reduce the hydraulic losses with an optimal blade outlet angle that maximizes the centrifugal pump head. Yuan et al. [16] studied the effect of blade outlet angle on chemical pumps to show that properly increasing the blade outlet angle improves the head and the efficiency. Other studies [17,18,19,20] have analyzed the effects of blade outlet angles, blade numbers, and the Stodala slip coefficient on the performance of centrifugal pumps. Fard et al. [21] tested the centrifugal pump performances with different blade outlet angles when handling water and viscous oils as Newtonian fluids. The results show that when the outlet angle increases, the centrifugal pump performance handling viscous fluids improves. Mohammadi et al. [22] studied the effects of blade outlet angle on the performance of multi-pressure pumps. The numerical and experimental analyses revealed that the maximum pump head and efficiency were witnessed at the outlet angle of 30°. Shi et al. [23,24] studied the effects of pre-swirl on a centrifugal pump without overload. Numerous studies have used the numerical simulation method to study the effects of different parameters on the performance and other fluid machinery [25,26,27,28,29].

As one of the important impeller design parameters, the blade outlet angle has a large influence on the internal flow, the performance and the overload characteristics of pumps. Therefore, this study analyzes the internal flow characteristics and the conditions that will limit overload of a low specific speed submersible pump for various blade outlet angles without changing the other geometric parameters of the impeller.

## 2. Geometry and Numerical Methods

The design flow conditions of a QY10-165 (6)-11 high-head no-overload submersible centrifugal pump are a rated flow rate Q_des_ = 10 m^3^/h, head = 165 m, rotating speed *n* = 2900 r/min, specific speed *n*_s_ = 46.46, and rated motor power *P* = 11 kW. The main structural parameters of the impeller are an impeller inlet diameter D_j_ = 53.5 mm, impeller outlet diameter D_2_ = 152 mm, impeller outlet width b_2_ = 6.5 mm, and blade wrap angle φ = 115°. The main structural parameters of the guide vane are a base circle diameter D_3_ = 156 mm and a guide vane inlet width b_3_ = 11 mm.

### 2.1. Impeller Design 

The design constraints for an impeller that will not experience overload give the basic impeller parameters listed in Table 1.

**Figure 1:**
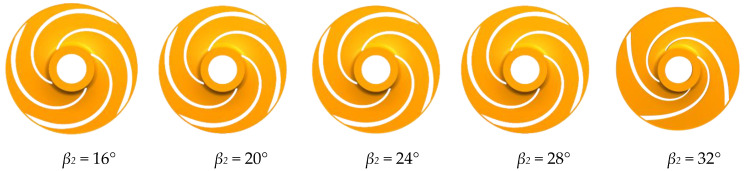
Impeller flow domain for the different blade outlet angles.

This study considered four impellers with five different blade outlet angles, β_2_ (16°, 20°, 24°, 28° and 32°) with the flow field and performance of the low specific speed centrifugal pump simulated for flow rates of 0.4Q~2Q. The impeller model is shown in Figure 1.

### 2.2. Modeling and Numerical Setting

**Figure 2:**
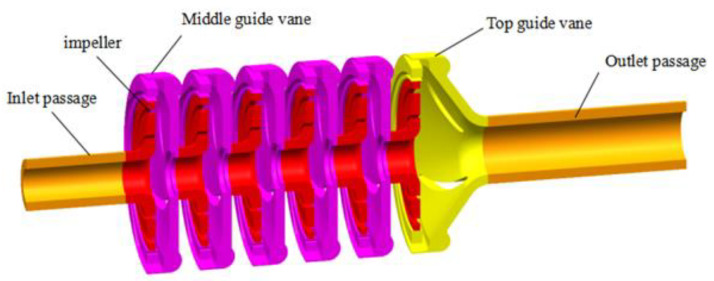
Pump flow domain model.

The impeller, radial guide vanes and inlet and outlet extensions of the QY10-165 (6)-11 pump were modeled in a three-dimensional model as shown in Figure 2. The flow in the impeller, guide vane and inlet and outlet extension sections was modeled numerically.

The model used an unstructured tetrahedral mesh. The large number of elements then required significant computing resources so more elements are not necessarily the best way to get a solution [30]. A grid independence study was conducted to ensure the calculational accuracy with a reasonable calculational efficiency using five meshes (with average element sizes of approximately 4, 3.5, 3, 2.5, and 2 mm) for the pump model. The predicted pump head, efficiency and power listed in Table 1 were used to evaluate the influence of the number of elements on the results. The results in Table 2 show that for these 5 meshes, the heads varied by less than 0.7%, the efficiencies varied by less than 1.5%, and the power varied by less than 3.6%. The variations for element sizes less than 3 mm were very small, so the 3 mm mesh was used for the calculations.

In this paper, ANSYS-CFX software was employed to calculate the steady flow field. The standard k-ε model was used as the turbulence model with the SIMPLEC algorithm used for the velocity–pressure coupling [31]. The water region in the impeller was a rotating domain, while the rest was static with a moving interface between them. The inlet boundary condition was the given pressure, with a reference pressure of 0 Pa. The outlet boundary condition was a specified mass flow rate. The wall surfaces were all no-slip. The convergence accuracy was 1.0 × 10^−4^.

## 3. Comparison of the Predicted and Experimental Results

**Figure 3:**
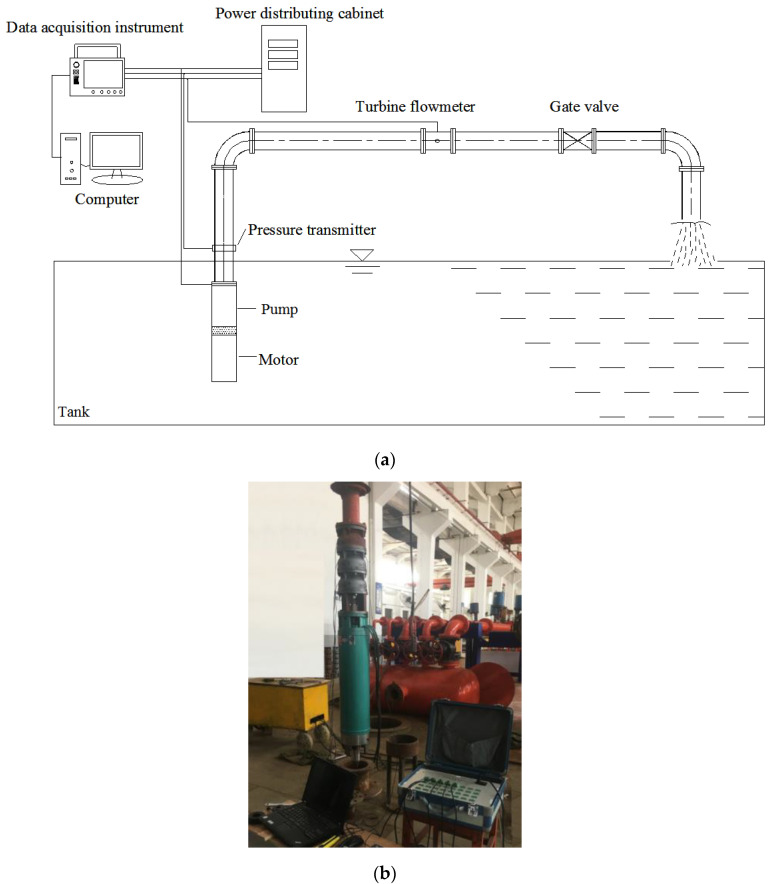
Submersible pump test rig: (**a**) schematic diagram; and (**b**) test site.

The pump performance was measured in a laboratory equipped with an automatic test system. Since the motor and pump were immersed in water, the motor output power (i.e., shaft power) could not be directly measured by a torque meter or dynamometer. Therefore, the motor loss analysis method was used to calculate the shaft power using a no-load test to determine the continuous loss [32,33]. The schematic diagram and test site of the pump test rig is shown in Figure 3.

**Figure 4:**
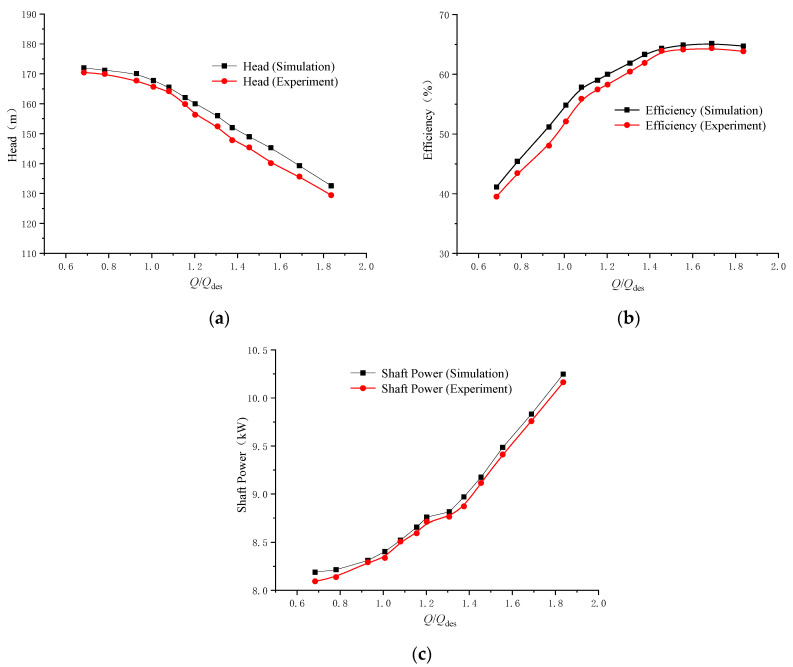
Predicted and measured pump heads, efficiencies and power: (**a**) flow-head curve; (**b**) flow-efficiency curve; and (**c**) flow-shaft power curve.

The predicted and measured pump performance characteristics are shown in Figure 4. The simulation results agree well with the experimental results with the predicted heads, efficiencies and power levels only slightly larger than the measured data. At the rated operating point, the predicted head was 0.6% higher, the efficiency was 1.2% higher and the shaft power was 0.8% higher.

## 4. Pump Flow Fields with Various Blade Outlet Angles

### 4.1. Effect of Blade Outlet Angle on the Pump Performance

If there is no rotation at the inlet, the theoretical pump head is:

(1) $$H_{t}=\frac{u_{2}}{g}(\upsilon_{u2})=\frac{u_{2}}{g}(\sigma u_{2}-\frac{\upsilon_{m2}}{\tan\beta_{2}}),$$

where *H_t_* is the theoretical head, m; *u*_2_ is the peripheral speed at the impeller outlet, m/s; *v*_u2_ is the component of impeller outlet velocity in the circumferential direction, m/s; *g* is the acceleration of gravity, m/s^2^; *σ* is the Stodora slip coefficient; and *v*_m2_ is the axial component of the absolute speed at the outlet of the impeller, m/s.

Increasing *β_2_* bends and shortens the flow channels between the blades. In addition, the diffusion angles between adjacent blades increase, which increases the hydraulic losses. Increasing *β_2_* also increases the absolute speed at the impeller outlet, *v_2_*, the circumferential component of the absolute velocity at the impeller outlet, *v_u2_*, the dynamic head, and the hydraulic loss in the impeller and the entire pump. In addition, when *β_2_* is too large, the flow-head curve is prone to forming a hump, which makes the operation unstable. Finally, when the other parameters remain unchanged, the pump shaft power increases with increasing *β_2_*, which can lead to overloading.

### 4.2. Effect of Blade Angle on Preventing Overload in a Centrifugal Pump 

**Figure 5:**
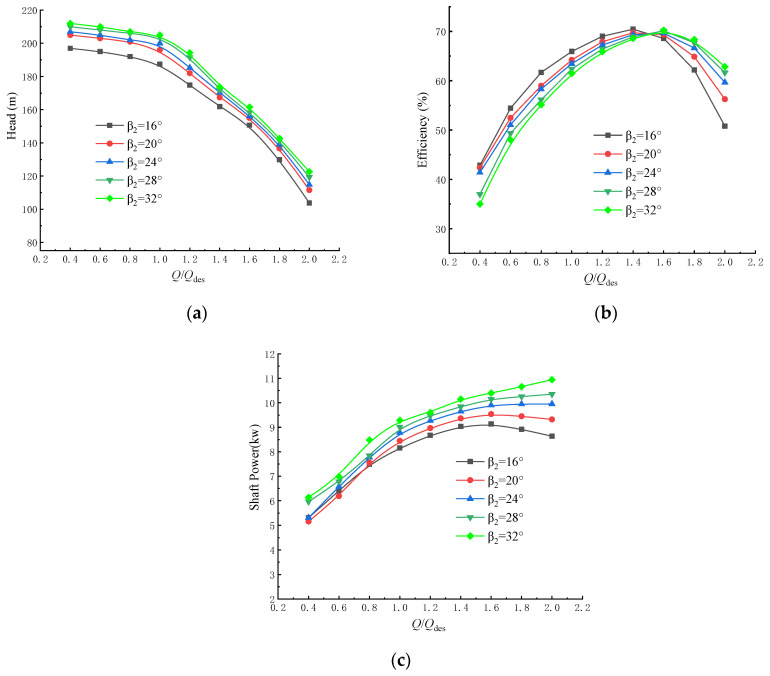
External characteristics of a centrifugal pump with various blade outlet angles: (**a**) flow-head curve; (**b**) flow-efficiency curve; and (**c**) flow-shaft power curve.

Figure 5 shows the influence of the various blade outlet angles on the external characteristic curves of the centrifugal pump. The flow-head curve shows that increasing the blade outlet angle gradually increases the head because increasing the blade outlet angle increases *v_u2_*. The basic pump equation then shows that the theoretical pump head will increase. When the blade outlet angle is reduced to 16°, the head drops sharply due to the large flow area. The flow-efficiency curve shows that for small flow rates (0–0.6Q_des_), the pump efficiency decreases with increasing blade outlet angle because increasing the blade outlet angle increases the channel curvature and shortens the channel. An increasing blade outlet angle also causes the flow passage to more quickly widen which also increases the hydraulic losses and reduces the efficiency.

The flow-shaft power curve shows that reducing the blade outlet angle gradually reduces the axial power of the centrifugal pump over the entire flow range. However, the blade outlet angle of 16° creates a turning point in the power curve for 1.6Q_des_. Smaller blade outlet angles will reduce the probability of overloading by having a maximum power point in the power curve.

### 4.3. Flow Field Analysis for Various Blade Outlet Angles

#### 4.3.1. Pressure Distribution Characteristics

**Figure 6:**
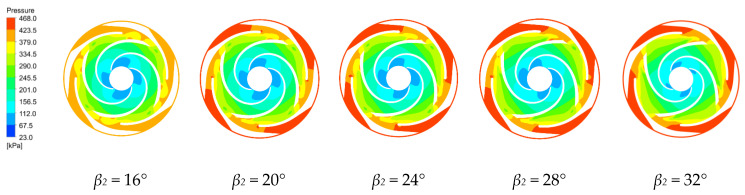
Pressure distribution along the first stage impeller for various blade outlet angles.

**Figure 7:**
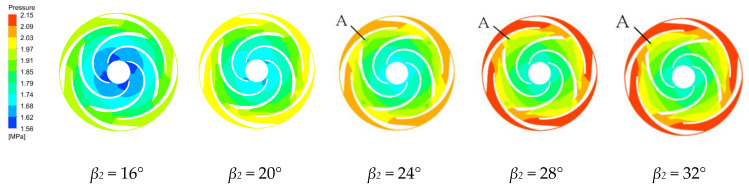
Pressure distribution along the final impeller for various blade outlet angles.

The pressure distributions along the middle section of the first and last stage impellers and guide vanes are shown in Figure 6 and Figure 7 for the four blade outlet angles at the rated working condition. The results show that the pressure distributions in these regions are quite similar. The hydrostatic pressure continuously increases from the blade inlet to the outlet and the pressure along the blade working surface is greater than the pressure along the back of the blade at the same radius. The high and low pressure interface in the impeller passage is very clear, because the static pressure of the fluid increases as the rotating blade works on the fluid. There is also a high and low pressure interface between the guide vane and the impeller. The guide vane has no obvious high and low pressure interface in the flow passage because the guide vane does no work on the fluid, but converts part of the kinetic energy of the fluid into pressure energy in the higher pressure area [34].

The pressure is relatively low at the first stage impeller inlet. Increasing the blade outlet angle increases the low pressure area at the impeller inlet, which increases the cavitation probability. Therefore, the *β_2_* = 16° design is less likely to cavitate. In the last stage impeller, the blade outlet angles of 24°, 28° and 32° create a local low pressure region at a region between the impeller and the guide vane, which means that a vortex has been generated there with backflow which increases the hydraulic losses. In the last stage impeller, increasing the blade outlet angle gradually reduces the low pressure area at the impeller inlet. The pressure distribution along the last stage impeller is more uniform than along the first stage impeller because the guide vane makes the flow more uniform.

#### 4.3.2. Velocity Distribution Characteristics

**Figure 8:**
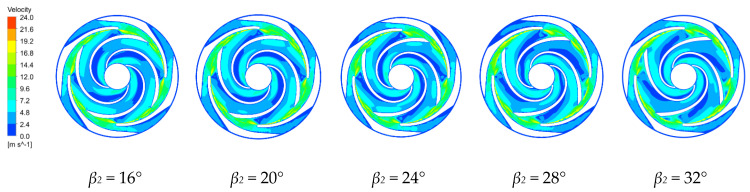
Velocity distributions along the first stage impeller for various blade outlet angles.

**Figure 9:**
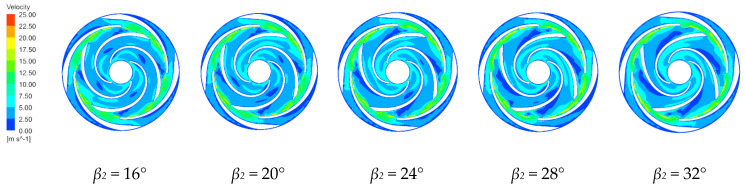
Velocity distributions along the final impeller for various blade outlet angles.

Figure 8 and Figure 9 show the velocity distributions along the first stage impeller and the middle section between the last stage impeller and the guide vane for the four blade outlet angles at the rated conditions. The velocity distributions in the centrifugal pump are similar for the four blade outlet angles. The impeller increases the fluid velocity along the passage, with non-uniform velocities across each passage. The low velocity region in the impeller flow area is due to the interference between the impeller and the guide vane, which forms a low-pressure vortex between the impeller outlet and the guide vane inlet. The velocity decreases from the inlet to the outlet of the guide vane region with the minimum velocity at the outlet because the expansion within the guide vanes gradually converts the fluid kinetic energy entering the guide vanes into pressure energy.

In the first stage impeller, the speed along the back of blade is generally lower than along the working surface. Increasing the blade outlet angle gradually increases the low-speed area along the working surface of the impeller while reducing the low-speed area along the back of the impeller. The impeller passage has a high-speed region with the fluid then forming a long strip of high speed in the guide vane passage, with this strip gradually increasing with increasing blade outlet angle. In the last stage impeller, blade outlet angles of 16° and 20° create a local low speed zone in the middle of the impeller flow channel that disappears at larger blade outlet angles. The high-speed zone at the blade outlet increases with increasing blade outlet angle, but the increases are not large. The velocities in the channel of the last stage impeller are more uniform than in the first stage. The velocities in the impeller passage are most uniform and the internal flow is most stable for *β_2_* = 16°.

#### 4.3.3. Turbulent Kinetic Energy and Velocity Vector Distributions

**Figure 10:**
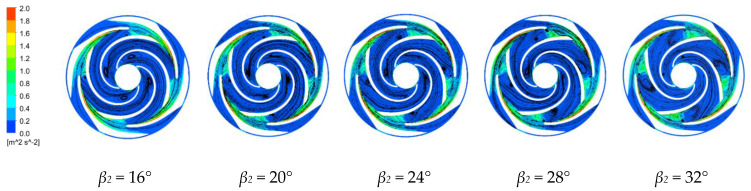
Turbulent kinetic energy and velocity vector distributions along the first stage impeller for the four blade outlet angles.

**Figure 11:**
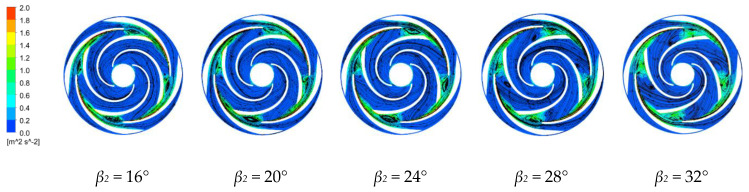
Turbulent kinetic energy and velocity vector distributions along the final stage impeller for the four blade outlet angles.

Figure 10 and Figure 11 show the turbulent kinetic energy and velocity vector distributions along the middle section of the first-stage impeller and along the last-stage impeller and the guide vane for the four blade outlet angles at the rated operating conditions. The turbulent kinetic energy is half of the product of the turbulent velocity fluctuations of the fluid and its mass [35]. The turbulent kinetic energy indicates the magnitude of the fluid turbulence. The uniformity of the turbulence distribution reflects the number of vortices in the fluid and the amount of viscous dissipation. A larger turbulent kinetic energy indicates larger turbulent vortices [36]. Flow separation does not necessarily occur in areas with large turbulent kinetic energies, but occurs in areas with large rates of change in the turbulent kinetic energy and mainly occurs near the blade working surface. The generated vortex rotates in the same direction as the direction of rotation. At the impeller outlet and the guide vane inlet, the turbulent kinetic energy is longer along the guide vane wall due to the higher fluid velocities at the impeller outlet and the impact on the guide vane wall. Increasing the blade outlet angle increases the flow separation along the blade working surface and the size of the axial vortex along the blade working surface, which rotates in the opposite direction to the direction of rotation of the impeller, and extends toward the impeller inlet.

Increasing the blade outlet angle increases the vortex near the working surface along the first-stage impeller due to the gradually widening of the flow path and the blade restricting the fluid motion. The frequency and intensity of the fluid impacting the wall surface are greater than with the smaller blade outlet angle. There is also a vortex along the back of the impeller. The flow has a large outlet speed from the impeller, with the energy then dissipated when the fluid enters the guide vane. In the last stage impeller, the slowing of the fluid through the guide vane greatly reduces the turbulent kinetic energy compared to that in the first stage impeller. The vortex in the impeller flow channel is smallest and the internal flow is more stable for *β_2_* = 16°.

## 5. Conclusions

The steady flow in a multi-stage submersible electric pump was modeled numerically for five different blade outlet angles. The effects of the blade outlet angle on the flow in the multi-stage submersible pump were analyzed in terms of the pump head, efficiency and power, the internal flow field, and the conditions that will prevent overloading. This study may provide guidance for the optimal design of small size pumps to prevent motor overload and to increase pump reliability, even for some micro-pumps. The results show that:

(1) The head gradually increases as the blade outlet angle increases. A smaller blade outlet angle gives a steeper head curve, which indicates more severe flow conditions. Proper reduction of the blade outlet angle can make the power decrease at higher flow rates so that overloading cannot occur.
(2) The pressure is low at the first-stage impeller inlet. Increasing the blade outlet angle significantly increases the low-pressure area of the impeller inlet, which increases the chance of cavitation, so the *β_2_* = 16° design has the lowest cavitation rate.
(3) Increasing the blade outlet angle increases the flow separation along the blade working face and increases the axial vortex along the blade working surface; the vortex rotates in the direction opposite to the impeller rotating direction, with the vortex extending to the inlet of the impeller.

## Figures and Tables

**Table 1:** Pump geometric parameters.

Design Parameters	Value
Rotating speed	2900 r/min
Design flow rate	10 m^3^/h
Head	165 m
Inlet diameter	54 mm
Outlet width	6.5 mm
Number of blades	4
Impeller outer diameter	152 mn
Blade wrapping angle	180°
Stages number	6

**Table 2:** Predicted pump performance for different meshes.

Number of Elements	Head (m)	Efficiency (%)	Power (kW)
1,546,562	166.36	58.62	8.3
2,412,168	166.21	58.41	8.2
3,451,566	165.52	57.85	8.4
4,313,151	165.43	57.75	8.7
5,255,682	165.46	57.78	8.6
